# The taper of cast post preparation measured using innovative image processing technique

**DOI:** 10.1186/1472-6831-10-19

**Published:** 2010-08-04

**Authors:** Khaled Q Al Hamad, Faruq A Al-Omari, Ahmad S Al Hyiasat

**Affiliations:** 1 Department of Prosthodontics, Faculty of Dentistry, Jordan University of Science & Technology, Irbid, Jordan; 2 Hijjawi Faculty for Engineering Technology, Yarmouk University, Irbid, Jordan; 3 Faculty of Graduate Studies, Jordan University of Science and Technology, Irbid, Jordan

## Abstract

**Background:**

No documentation in the literature about taper of cast posts. This study was conducted to measure the degree of cast posts taper, and to evaluate its suitability based on the anatomy aspects of the common candidate teeth for post reconstruction.

**Methods:**

Working casts for cast posts, prepared using Gates Glidden drills, were collected. Impressions of post spaces were made using polyvinyl siloxan putty/wash technique. Digital camera with a 10' high quality lens was used for capturing two digital images for each impression; one in the Facio-Lingual (FL) and the other in the Mesio-Distal (MD) directions. Automated image processing program was developed to measure the degree of canal taper. Data were analyzed using Statistical Package for Social Sciences software and One way Analysis of Variance.

**Results:**

Eighty four dies for cast posts were collected: 16 for each maxillary anterior teeth subgroup, and 18 for each maxillary and mandibular premolar subgroup. Mean of total taper for all preparations was 10.7 degree. There were no statistical differences among the total taper of all groups (P = .256) or between the MD and FL taper for each subgroup. Mean FL taper for the maxillary first premolars was lower significantly (P = .003) than the maxillary FL taper of the second premolars. FL taper was higher than the MD taper in all teeth except the maxillary first premolars.

**Conclusions:**

Taper produced did not reflect the differences among the anatomy of teeth. While this technique deemed satisfactory in the maxillary anterior teeth, the same could not be said for the maxillary first premolars. Careful attention to the root anatomy is mandatory.

## Background

There has been a general consensus that endodontically treated teeth are brittle and subjected to fracture [1-4]. These teeth are usually associated with extensive loss of tooth structure due to caries,, trauma, or further endodontic treatment. Similar results were reported in relation to the removal of dentin during root canal preparation for metal posts;the increase in diameter of posts did not provide a significant increase in retention, however, it could increase the stiffness of posts at the expense of the remaining dentine and the fracture resistance of the root[5-12].

Retention of posts often requires tooth structure removal; a procedure that may reduce the strength of roots. Cast tapered posts were found the least retentive in all post designs [8]. These comparisons are relevant only if the post fits the root canal accurately, because retention is proportional to the total surface area. When the canal shape is ovoid, the walls of prefabricated posts are unlikely to adapt well along their entire interface with the canal walls. As a result, the post may not fit the preparation closely, and the luting agent may not totally fill the interface. This makes parallel sided posts only effective in the most apical portion of the post space because of the considerable flare of the post space in the coronal portion [13].

Pettiette et l.,[14] evaluated the effect of various tapers of canal preparation on the retention of posts. The canals were prepared using NI TI instrument of tapers ranging from 0.04 to 0.12-mm instrument taper and were compared for retention against canals prepared using 0.02-mm taper instruments. Canals prepared with 0.04 taper instruments provided the best retention, while 0.02 taper size showed the worst retention. The authors concluded that the difference in post retention was related to the differences in film thicknesses. The thinner the film thickness the better the retention of the post; however, too little cement film thickness did not adequately maintain proper adhesive properties. On the other hand, tapers that produced too divergent canals allowed thick cement film to be present, which resulted in intercement bond failure.

Several methods for post preparation were investigated, including rotary instruments, heated instruments and solvents. The literature is equivocal on post space preparation and no method has been found consistently superior. When the mechanical method is preferred, it has been established that Gates Glidden^® ^drills and P-type^® ^reamers used on low-speed are the safest instruments [3].

It was stated that round canals, particularly in maxillary central incisors, can be prepared to provide a post space with parallel walls or minimal taper [13]. Conversely, canals with elliptical cross sections must be prepared with a restricted amount of taper. Taper of 6-8 degrees were reported to be necessary to ensure adequate retention while eliminating undesired undercuts [13]. This is analogous to extracoronal preparations; retention increases rapidly as vertical wall taper is reduced.

Although several advances in canal preparation were introduced recently, canal preparation using Gates Glidden^® ^drills technique is still the standard technique taught at the dental school of the Jordan University of Science and Technology, and is still common in Jordan and other parts of the world.

There is no documentation in the literature on the taper of post space. This study was conducted to measure the degree of post space taper of cast posts, which were prepared using Gates Glidden^® ^drills technique, using innovative image program. Also to evaluate the relation and suitability of this technique based on the anatomy aspects of the common candidate teeth for post reconstruction.

## Methods

The research was performed with the approval of the relevant research committees in the department and faculty of dentistry in the Jordan University of Science and technology.

**Figure 1:**
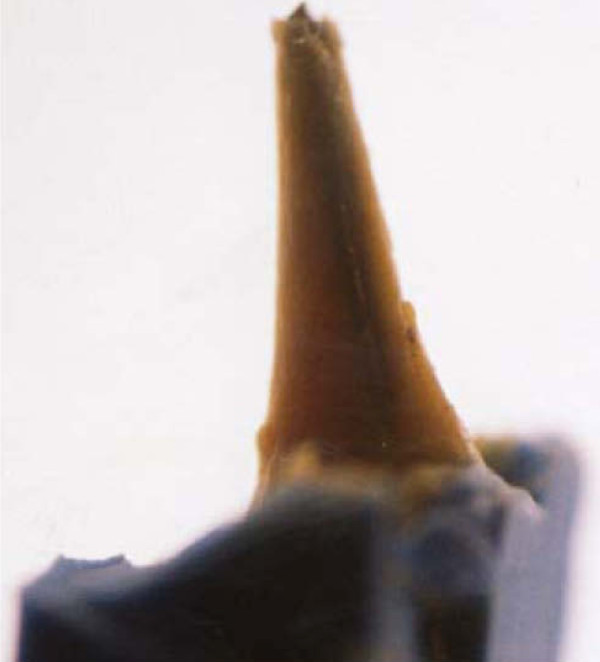
**A typical acquired image in the MD Dimension for a maxillary central incisor**.

Working casts of post crown cases, which were treated by 5^th ^year dental students, were collected from the dental laboratory at the Dental Teaching Center of JUST. These cases included only the maxillary anterior teeth group and the maxillary and mandibular premolar teeth group. Dental students followed strictly the instructions on post preparations given by two clinical supervisors; who met and agreed on the technique before starting the study. The technique involved post preparations using simultaneous Gates Glidden^® ^drills to one size beyond the largest file size used for endodontic treatment. Only working casts for patients who received successful endodontic treatment by dental students at the dental health centre were selected. A sectional impression of each post space was taken using a polyvinyl siloxan^® ^putty/wash impression technique (3 M ESPE^®^). The wash impression material was injected into the canal using A lentulo spiral^® ^drill along with a stainless steel wire. The wire was modified by making a loop at one end and the wire was roughened and tray adhesive (3 M ESPE tray adhesive^®^) was added. The part of the impression, which was related to the post space, was cut out of the impression and then was labeled with the correspondent tooth, Figure 1. Sony^® ^XCL-U1000 camera with a 10 × special high quality attachment lens was used for capturing two digital images of each canal space specimen; one for Facio-Lingual dimension (FL) and the other for the Mesio-Distal (MD) dimension. Each canal impression was placed on a radiology viewer box facing the digital camera, which was held on a stand in a position perpendicular to the line of sight from the canal impression position. Two experienced dentists watched and supervised the image acquisition process. Two specimens were randomly selected to serve as a control group; during the process of capturing the images of the specimens, the control group specimens were remounted for imaging in every 10 studied specimens. The images collected for the control group were processed independently to validate the accuracy and reproducibility of the image acquisition process. Duplicate measurements were obtained to measure the reliability of the examination using percent agreement, Kappa test, which revealed more than 95% agreement in the measurements of the control group.

The size of the captured images was 1365 × 741 pixels with a pixel spacing of 0.0125 mm/pixel. Figure 1 presents an example of an acquired image.

### Image Processing [15]

**Figure 2:**
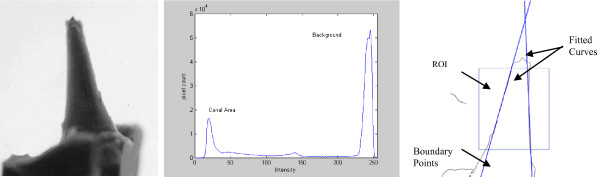
**(a) Grayscale image, (b) its corresponding histogram, and (c) Processed image after localizing taper boundary points and curve fitting**.

The first step in the process was to convert the colored image into grayscale image. After that, the grayscale image was converted to binary image by thresholding in order to isolate the canal impression from the background. The output binary image had values of 0 (black) for all pixels in the input image with luminance less than a threshold value, T, and 1 (white) for all other pixels. The threshold value T was selected based on the histogram of the grayscale image, which was bimodal in this case. Therefore, T was selected halfway between the main two peaks in the histogram, as shown in Figure 2. To eliminate the effect of any unwanted pixels, morphological cleaning was performed on the binary image. According to this operation, any 1-pixel that was surrounded by zero pixels was wiped out. As a result of this process, the binary image object representing the canal impression was identified and isolated from the background. The next step in the process was to allow interaction with the user, who had to identify the region on the tapered canal impression at which the taper angle was to be measured. This included the area between the start of the canal coronally to the end of the apical area. The user drew a polygon surrounding the tapered region using the computer mouse. Based on this selection, a mask image was created to exclude everything in the binary image except for the region of interest (ROI). The boundary of the object enclosed by the ROI was found using two morphological operators; namely erosion and dilation. An eroded image was subtracted from a dilated image resulting in a boundary image containing only the boundary of the region of interest. The ROI was then scanned and the coordinates of each boundary point representing each side of the canal impression was recorded. Two coordinate vectors, V_1 _and V_2 _were extracted, in which each entry in these vectors represented the row and column position of the corresponding boundary point in the image. Each vector was used to fit its corresponding entries to the best linear curve using auto-regression techniques.

### Linear Regression Curve Fitting [15]

For fitting a set of data points to a straight line, an expression of the form *y *= *mx *+ *b *was used, where *m *was the line's slope and *b *was its *y*-intercept. Linear regression techniques were used to estimate the slope and *y*-intercept in such a way to minimize the Mean Square Error (MSE). The MSE was defined as the sum of the squares of the deviations from the mean. Assume that the *x*_i _values were precise and all uncertainty is in *y*_i _. The deviations in *y*_i _were given by

(1) $$e_{i}\;=\;y_{i}\;-\;\left(m\;x_{i}\;+\;b\right)$$

Then, the MSE was given as:

(2) $$MSE\;=\;\sum_{N}{\left(e_{i}\right)}^{2}\;=\;\sum_{N}{\left(y_{i}\;-\;\left(m\;x_{i}\;+\;b\right)\right)}^{2}$$

Where, *N *is the number of (*x*_i_,*y*_i_) pairs. To minimize the MSE, the expression was derived with respect to m and *b *was set that equal to zero:

(3) $$\begin{array}{ccc} \frac{\partial MSE}{\partial m}\;=\;0, & and & \frac{\partial MSE}{\partial b}\;=\;0 \end{array}$$

Solving the above two equations given in (3) with respect to *m *and *b *gave

(4) $$m\;=\;\frac{N\;\left(\sum_{N}x_{i}\;y_{i}\right)\;-\;\left(\sum_{N}x_{i}\right)\;\left(\sum_{N}y_{i}\right)}{N\;\left(\sum_{N}x_{i}^{2}\right)\;-\;{\left(\sum_{N}x_{i}\right)}^{2}}$$

and,

(5) $$b\;=\;\frac{\;\left(\sum_{N}x_{i}^{2}\right)\;\left(\sum_{N}y_{i}\right)-\;\left(\sum_{N}x_{i}\right)\;\left(\sum_{N}x_{i}\;y_{i}\right)}{N\;\left(\sum_{N}x_{i}^{2}\right)\;-\;{\left(\sum_{N}x_{i}\right)}^{2}}$$

The angle between two lines,θ, with slopes *m*_1 _and *m*_2 _was then given as

(6) $$\theta \;=\left|\;\tan^{-1}\;\left(m_{1}\right)\;-\;\tan^{-1}\;\left(m_{2}\right)\right|$$

where tan^-1^( ) is the inverse tangent, and |.| was the absolute value. Figure 2c showed the two fitted curves imposed on the processed image.

## Results

A total of 84 working dies for cast post fabrications were collected: 48 dies for the maxillary anterior teeth group, 18 dies for the maxillary premolar groups and 18 dies for the mandibular premolar groups. The maxillary anterior group was subdivided into central, lateral, and canine subgroups. Premolar groups were also subdivided into first and second premolars subgroups in both the maxillary and mandibular premolar groups. Two images were acquired for each canal specimen in the FL and MD dimensions for a total of 168 images. The acquired images were then processed, as discussed before, and the taper for each impression was evaluated. The data were analyzed using Statistical Package for Social Sciences software (version 15.0: SPSS Inc., Chicago, IL, USA.) and One way Analysis of Variance (ANOVA) to draw statistical inferences. The means and standard deviations for each group and subgroup were calculated in the MesioDistal (MD) and FacioLingual (FL) dimensions, as presented in Table 1. The total mean of taper for all preparations was 10.7 degree. The highest mean of total taper was registered in the maxillary premolar group, followed by the mandibular premolars, and last by the maxillary anterior teeth group.

**Table 1:** Means (μ) and standard deviations (SD) of canal taper for the studied teeth.

		MD Taper		FL Taper		Total Taper	
		
	N	μ	SD	μ	SD	μ	SD
**Maxillary anterior teeth**	**48**	**8.32**	**2.46**	**9.45**	**3.63**	**8.89**	**4.2**

Maxillary central incisors	16	8.50	3.54	10	4.66	9.25	4.05

Maxillary lateral incisors	16	7.81	1.44	9.79	4.94	8.80	5.54

Maxillary canines	16	8.48	1.45	8.59	3.80	8.53	2.75

**Maxillary Premolars**	**18**	**11.31**	**5.61**	**10.28**	**3.45**	**10.80**	**7.87**

Maxillary first premolars	9	10.69	8.51	7.27	1.13	8.98	5.87

Maxillary second premolars	9	11.93	2.23	13.29	1.48	12.61	7.2

**Mandibular Premolars**	**18**	**8.38**	**1.54**	**10.20**	**3.39**	**9.29**	**4.54**

Mandibular first premolars	9	7.94	1.34	9.33	3.56	8.64	4.1

Mandibular second premolars	9	8.82	1.87	11.06	3.71	9.94	4.45

**Figure 3:**
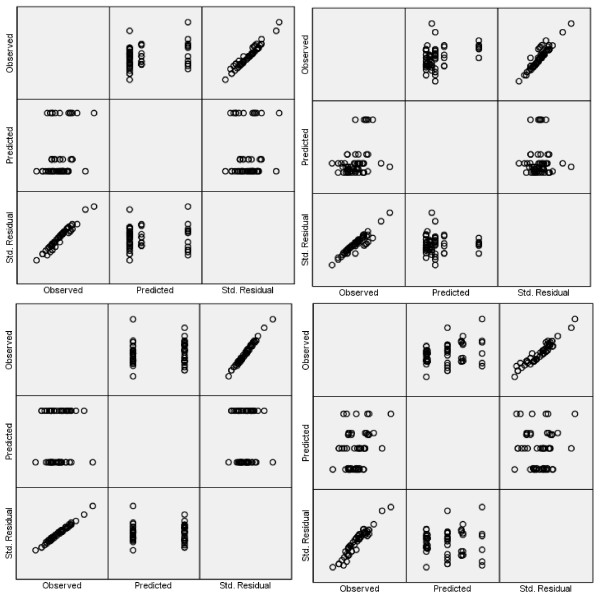
**Normal quantile plot of the calculated taper with respect to (a) teeth groups, (b) subgroups, (c) imaging dimension, and (d) both groups and imaging dimension together**.

The validity of assumptions underlying ANOVA was verified, in order to draw statistical inferences on the evaluated tapers. Normal quantile plot was carried out in order to check for normality of the measured taper with respect to teeth group and imaging dimension. Figure 3 showed the normal quantile plot when the calculated taper was set as the dependent variable while the teeth group (Figure 3a), subgroup (Figure 3b), imaging dimension (pose) (Figure 3c), as well as teeth group and pose simultaneously (Figure 3d) were set as independent variables, respectively. The plots in the lower left corner of each figure depict the ordered standardized residuals on the vertical axis and the normal quantile values on the horizontal axis. The residuals are fairly linear with respect to quantile values, which in turn validate the normality of the data. To check for homogeneity, Levene's test of equality of Error Variance was performed. This test checks the validity of the null hypothesis that the error variance of the dependent variable is equal across different groups. When performed, it was found that there was no significant difference between the variances. Henceforth, the data was reasonably assumed to be homogeneous. Finally, each specimen represents a different working die that was prepared independently from other working dies. Furthermore, each image was processed independently to measure the taper providing no clue as to the likely value of other tapers. Therefore, it was practically assumed that taper values were statistically independent.

In order to study the effect of the three main independent variables namely; tooth group, subgroup, and image dimension (pose), one-way ANOVA was conducted several times taking into account the effect of these variables in the taper value. Table 2 presented the value of the significance (*p*) for each case. As can be seen from the table, ANOVA for total taper revealed no significant differences among the groups (*p *= 0.256).

**Table 2:** ANOVA results with Taper as dependent variable

Factor(s)	Dataset Processed	Significance (P)
Groups (Both Poses)	168	0.256
Groups (Pose = MD)	84	0.156
Groups (Pose = FL)	84	0.842

Subgroups (Group I, Both Poses)	96	0.857
Subgroups (Group I, Pose = MD)	48	0.905
Subgroups (Group I, Pose = FL)	48	0.789
Subgroups (Group II, Both Poses)	36	0.169
Subgroups (Group II, Pose = MD)	18	0.819
Subgroups (Group II, Pose = FL)	18	0.003 *
Subgroups (Group III, Both Poses)	36	0.426
Subgroups (Group III, Pose = MD)	18	0.544
Subgroups (Group III, Pose = FL)	18	0.592
Subgroups (All Groups, Pose = MD)	84	0.699
Subgroups (All Groups, Pose = FL)	84	0.461

Poses (Group I)	96	0.307
Poses (Group II)	36	0.710
Poses (Group III)	36	0.260

* indicates statistical significance

Group I: Maxillary anterior teeth

Group II: Maxillary Premolars

Group III: Mandibular Premolars

With regard to the maxillary anterior teeth, the central incisors had the highest mean of total taper (9.25), followed by the lateral incisor (8.80), and the canine group (8.65). The mean of total taper for maxillary and mandibular second premolars were higher than the maxillary and mandibular first premolars, Table 1. The differences between the subgroups were not statistically significant, Table 2.

The maxillary central incisors had the highest MD an vd FL taper in the maxillary anterior group. The lowest MD taper was registered in the lateral incisor group, while the canine group had the lowest FL taper. No significant differences were detected between the subgroups in both the MD dimension (*p *= 0.905) and the FL dimension (*p*= 0.789), as reported in Table 2.

On the other hand, the mean taper for the maxillary first premolars was lower than the maxillary second premolars in both the MD and FL dimension. According to Table 2, the difference in the FL dimension was statistically significant (*p *= 0.003). The same was true in the mandibular groups in both dimensions, but with no statistically significant differences. Finally, The FL taper for all teeth was higher than the MD taper except in the maxillary premolar group.

## Discussion

This study included three groups for investigation: the maxillary anterior teeth group (including 3 subgroups for the central, lateral, and canine teeth), and the maxillary and mandibular premolar groups (including first and second premolar subgroups). Molars and mandibular incisors were not included in the study. Cast posts are not routinely used in these teeth; mandibular anterior teeth have thin roots which make it difficult to prepare a post, and molars usually do not require posts because they have more tooth structure and large pulp chambers to retain a core.

The amount of remaining dentin and the nature of root morphology are important before attempting to prepare any canal space for post installation. Root diameter may differ in the FL and MD dimension. Maxillary central and lateral incisors usually have sufficient bulk of roots to accommodate post restorations. However, care must be exercised with post of excessive length if the roots taper rapidly to the apex. The outline and pulp cavities of these teeth are similar. Central incisors are larger, and it is extremely rare for these teeth to have more than one root or one canal. Where abnormalities do occur they seem to affect the maxillary lateral incisor, which may present with an extra root, second canal, dens invaginatus, germination, or fusion. The canal is tapered with an oval or irregular cross section cervically that becomes round only very near the apex. The root canal differs greatly in outline when viewed in FL or MD dimension. The former generally shows fine straight canals, while the MD dimension shows a wider canal [16]. After post preparation, the taper in the FL dimension for these teeth was larger than the MD pose, but with no statistical significance. This might indicate an over tapering and over preparation in the FL dimension.

The FL taper in the canine subgroup was smaller than the maxillary central and lateral incisors, and was larger, but not statistically significantly, than the taper of the canine group in the MD dimension. These results did not reflect the anatomy of the roots and root canals; the maxillary canines have wide faciolingual roots and root canal spaces. The root canal is oval and does not begin to become round until the apical third [16].

The maxillary first premolars normally have two separate canals, which are usually straight with a round cross section. The root canals are wide buccopalatally but narrow mesiodistally. This was not consistent with the results after post preparation, in which, the FL dimension was significantly smaller than the taper in the MD dimension. Increasing the taper is highly risky in such roots, because they present a variety of problems for post retained restorations. Root walls are commonly thin and roots taper rapidly to the apex. Proximal invaginations and canal splitting are common. Parallel sided posts might be more suitable in this group of teeth. The same observations are true for the maxillary second and mandibular premolars, but these teeth tend to have single canals and greater bulk of tooth structure. One area of concern with the maxillary first premolar is the angle of the crown to the root, often the root will be lingually inclined and active drilling of a post space perpendicular to the occlusal surface will result in a perforation along the facial wall of the root.

There was no documentation in the literature of similar studies for the purposes of comparisons. Pettiette et al., [13] used NI-Ti instruments to produce post space preparations of controlled tapers ranging from 0.04 to 0.12-mm. It was to not possible to compare the tapers reported in this study with those reported by Pettiette et al., [13] because teeth investigated in this study were root treated using the traditional step back technique, and the canals were prepared for posts using Gates Glidden^® ^drills.

The aim of this study was not to evaluate the performance of dental students due to lack of references for comparisons. The data reported were only descriptive and helped to draw conclusions on the safety of the technique used, based on the anatomy aspects of the teeth.

Only cases treated by undergraduate students were used in this study in order to provide the same conditions and technique as much as possible. The differences in canal tapers among the groups might be attributed to the differences in the skills of students rather than the technique used. All cases were performed in strict academic atmosphere and under close supervision in order to minimize this effect. Further studies using cases treated by professionals are recommended.

Post space preparation taper for all groups were higher than the recommended 6-8 degree taper. This recommendation was not based on in vitro or in vivo studies of post crown restorations; instead, it was solely made analogous to the extra coronal preparations. Recently, the recommendations for extra coronal preparations have been subjected to scientific scrutiny. It has been determined that dental students, general practitioners, and prosthodontists do not routinely create such minimal angles [17-21]. More recently, resistance to lateral forces and not retention along the path of insertion has been advocated as the determining factor in a crown's resistance to dislodgment. Resistance testing was more sensitive than retentive testing to changes in taper [22-24]. Shillingburg et al., [25] recently suggested that the taper of crown preparation should be between 10-22 degree. Similar recommendations were also suggested by Goodacre et al., [26]. Based on the available evidence; it was difficult to decide whether or not the results reported for post taper were satisfactory. Root canals have natural taper before endodontic treatment and when root treated, the taper is further increased. Despite the technique used, the final taper of the post space will be influenced by the taper produced after root canal therapy. The advent of rotary nickel-titanium instruments led to the possibility of rounder canal profiles and more controlled taper than the hand files [27,28]. Further investigations in this area are required.

Resistance to fracture is another important factor that must be achieved with post and core retained restorations. The mechanism of root fracture is still not fully understood. Root canal treatment was suggested as a factor influencing the incidence of vertical root fracture [29-31]. It has not been established whether fractures occur at the time of filling or manifest themselves at later time [32]. Rundquist & Versluis [33] studied the influence of different canal tapers on radicular stress distributions and reported that during root canal obturation, root stress decrease as the canal taper increase, while the relation is reversed after root filling is complete and occlusal load is applied. The authors also reported that vertical fractures initiated at the apex are a result of filling force, whereas vertical root fractures initiated cervically are a manifestation of subsequent masticatory events on the root filled tooth [33].

Oval-shaped root canals, which are found in approximately 25% of roots [34], pose problems with regard both to the effectiveness of canal preparation and to fracture susceptibility. The narrow radius of curvature at the buccal and lingual extensions of the canal means that these locations serve as sites of stress concentration [35]. A finite element study indicated that when an internal load was applied in models with a round canal, the stress distribution was low and relatively uniform. The thickness of the surrounding root dentine hardly affected this distribution. In contrast, the oval canal showed much higher stresses and a very uneven stress distribution [36].

There were no significant differences among the mean tapers of the groups despite the vast anatomic differences. This could be related to the fact that standard deviations of many groups/subgroups were too big compared to their respective means, which probably made it difficult to obtain statistical significant differences between teeth with different anatomy aspects. This could be related to the post preparation technique, step back technique used in endodontic therapy, or both. Intuitively, it could be still reasonable to speculate that the lack of statistical differences between the different subgroups was and indication of over tapering and over reduction of tooth structure, especially in the maxillary first premolar teeth. Increasing the taper of the canal preparation by removing more dentine from the canal wall would diminish the structural durability of the root and make them more susceptible to fracture [10,36].

To minimize failures, the optimum diameter for the tapered post of cast alloy relative to root diameter was reported to be approximately 1:4 [37]. Post fitting in oval canals was affected by different drill/tips used for canal preparation. A fine grit oval tip combined with oval posts was reported to provide the best post fitting [38].

Potential fracture might be reduced by practitioners being aware of risk factors such as the post preparation technique, post selection, coronal restoration, and inappropriate selection of tooth abutment for prosthesis [39].

This study provided a descriptive data on the taper of post space prepared using simultaneous Gates Glidden^® ^drills. No attempt was made to measure the remaining tooth structure, the retention/resistance to dislodgment, or the resistance of the posts to fracture. These are important features and required further studies.

The methodology for measuring taper was based on image processing techniques.. Unlike other studies, in which microscopic visual perception was utilized, this study used a fully automated innovative process to locate the taper region and hence measured the amount of taper with minimal human interaction. Such an approach guaranteed minimal inter- and intra-inspector variation.

## Conclusions

The technique used for post preparation did not follow the anatomy of the roots and root canals. No differences in post taper were found between the maxillary anterior teeth, maxillary premolars, or mandibular premolars despite the vast differences among the anatomy of these teeth. While using this technique might be satisfactory in the maxillary anterior teeth, the same could not be said for the maxillary first premolars.

The Innovative Image processing technique used in this study was valid for data processing in this field.

## Competing interests

The authors declare that they have no competing interest.

## Authors' contributions

AK participated in developing the methodology of the research, the clinical supervision of the selected cases, performed the experiment, and did the entire write up except the image processing technique part. AF performed the image processing technique section, the statistical analysis, and the relevant write up. HA provided the idea of this research, collected the cast and provided the required material for the experiment, and participated in the clinical supervision of the selected cases and developing the methodology of this research.

All authors have read and approved the final manuscript.

## Pre-publication history

The pre-publication history for this paper can be accessed here:

http://www.biomedcentral.com/1472-6831/10/19/prepub
